# Prospects for immunotherapy of depression based on cell technologies

**DOI:** 10.1192/j.eurpsy.2021.2022

**Published:** 2021-08-13

**Authors:** E. Markova, M. Knyazheva

**Affiliations:** Neuroimmunology Lab, State Research Institute of Fundamental and Clinical Immunology, Novosibirsk, Russian Federation

**Keywords:** modulated immune cells, Cell technologies, Depression, Immunotherapy

## Abstract

**Introduction:**

Cell technologies actively used in the treatment of many diseases. These technologies are based on manipulating the patient’s cells outside the body, as a result of which cells acquire a higher therapeutic potential.

**Objectives:**

No doubt the essential role of immune cells and their biologically active products in the pathogenesis of depression, which allows to view the modulated immune cells as model objects for developing new approaches to immunotherapy for depression.

**Methods:**

(CBAxC57Bl/6) F1 depressive-like male mice, developed under the long-term social stress, were undergoing the transplantation of syngeneic immune cells with in vitro caffeine-modulated functional activity. Recipient’s behavior, immune and nervous systems functional activity were studied.

**Results:**

It was found that immune cells isolated from depressive-like mice and treated in vitro with caffeine change their properties and after intravenous administration to syngeneic depressive-like recipients have a significant positive psycho- and neuroimmunomodulatory effects, affecting the main depression pathogenetic mechanisms: behavioral editing (reduction of anhedonia, stimulation of exploratory behavior and activity in the forced swimming test); hippocampal neurogenesis stimulation against the background of increased BDNF; modulation of cytokine production by brain cells, indicating a decrease in neuroinflammation; modulation of the immune system functional activity (stimulation of the immune response, splenocytes proliferation, reducing systemic inflammation, decrease spleen tryptophan catabolism).

**Conclusions:**

The results serve as an experimental substantiation of a fundamentally new approach to immunotherapy of depression based on the introduction of immune cells with functional activity modulated outside the body and open up the possibility of developing new methods of immunotherapy of depressive states in humans.

**Disclosure:**

No significant relationships.

